# Knowledge about systemic inflammatory response syndrome and sepsis: a survey among Dutch emergency department nurses

**DOI:** 10.1186/s12245-016-0119-2

**Published:** 2016-07-15

**Authors:** L. C. van den Hengel, T. Visseren, P. E. Meima-Cramer, P. P. M. Rood, S. C. E. Schuit

**Affiliations:** Department of Emergency Medicine, Erasmus Medical Center, Rotterdam, The Netherlands; Erasmus MC Zorgacademie: Training Center for Health Professionals, Erasmus Medical Center, Rotterdam, The Netherlands

**Keywords:** Sepsis, SIRS, Emergency department, ED, Knowledge, Nurses

## Abstract

**Background:**

Sepsis has a high mortality. Early recognition and timely treatment are essential for patient survival. The aim of this study is to examine the factors that influence the knowledge and recognition of systemic inflammatory response syndrome (SIRS) criteria and sepsis by emergency department (ED) nurses.

**Methods:**

A prospective, multi-center study including 216 ED nurses from 11 hospitals and academic medical centers in The Netherlands was conducted in 2013. A validated questionnaire was used to evaluate ED nurses’ knowledge about SIRS and sepsis. Questions about demographic characteristics were also included, to investigate factors that may contribute to the knowledge about SIRS and sepsis.

**Results:**

The mean total score was 15.9 points, with a maximum possible score of 29 points. ED nurses employed at hospitals with a level 3 intensive care unit (ICU) scored significantly higher than their colleagues employed at hospitals with a level 1 or 2 ICU. Recently completed education in sepsis was associated with a higher score. The employees in low ICU level hospitals who reported recent education did not score significantly lower than their ICU level 3 colleagues. ED nurses over the age of 50 scored significantly lower than their younger colleagues.

**Conclusions:**

The knowledge of ED nurses concerning SIRS and sepsis rises proportionally with the level of ICU in hospitals. Recent education in sepsis raises knowledge level as well. We recommend that when there is a low exposure rate to SIRS and sepsis, more emphasis should be placed on regular education.

## Background

Sepsis has a high mortality rate and contributes to high costs in healthcare [1, 2]. Rapid diagnosis and early treatment of patients with sepsis reduces the mortality significantly, thereby lowering costs [3–6].

Systemic inflammatory response syndrome (SIRS) criteria were published in 1992 and consist of several parameters such as body temperature higher than 38 °C or lower than 36 °C, heart rate higher than 90/min, respiratory rate higher than 20/min or PaCO_2_ lower than 32 mmHg and white blood cell count higher than 12,000 cells/μl or lower than 4000 cells/μl [7].

Several degrees of sepsis are defined: SIRS plus infection, ‘severe sepsis’ (sepsis associated with organ dysfunction, hypoperfusion or hypotension) and ‘septic shock’ (sepsis with arterial hypotension despite adequate fluid resuscitation). It is important to define the degree of sepsis and initiate appropriate antibiotic treatment because severe sepsis and septic shock have high hospital mortality rates [7].

When a septic patient arrives at the emergency department (ED), in most hospitals, he or she is first triaged by an ED nurse [8]. Early treatment of sepsis is only possible when sepsis is recognized promptly. Since nurses play a vital role in care of the septic patient, we would like to know what factors influence the knowledge of SIRS and sepsis in ED nurses. In a recent study, delay in diagnosis was the most reported barrier in the care of septic patients [9].

Previous studies have explored the knowledge of the SIRS criteria, along with the knowledge of the different stages of sepsis among medical school graduates and physicians [10–12]. There seems to be a lack of clarity and consistency in defining sepsis; this may contribute to delay in diagnosis and early treatment [10]. Furthermore, these studies lack large cohorts and validated questionnaires. Another study has shown poor knowledge about SIRS and sepsis in ward nurses (*n* = 73).

They recommended targeted education to achieve the Surviving Sepsis Campaign’s aim of reducing mortality due to sepsis [13]. However, there is little evidence to support this recommendation.

### Objectives

At this time, there is little understanding of what factors determine whether or not ED nurses recognize SIRS and the different stages of sepsis. It is expected that level of experience and training, regular education sessions, a clear sepsis protocol and hospital size may contribute to recognition and registration of SIRS and sepsis. When these factors are clearly recognized, a targeted educational intervention can be implemented.

### Goals of this investigation

The aim of this study is to examine the factors that influence the knowledge and recognition of SIRS criteria and sepsis by ED nurses.

## Methods

### Study design and setting

We conducted an observational multi-center study including ED nurses from 11 hospitals in the western part of The Netherlands. These hospitals include secondary (general) and tertiary (academic) healthcare centers. We evaluated knowledge about sepsis and SIRS by using a validated questionnaire. Data was collected in a 3-month time period (May to July 2013).

### Selection of participants and data collection

In The Netherlands, there are three different levels of intensive care units based on the nature of the facility, the care process and the clinical standards and staffing requirements. Level 1 is the smallest, most basic intensive care unit (ICU) and level 3 is the largest, most extensive ICU. Therefore, in The Netherlands, hospitals with a level 3 ICU often receive the most septic patients. Consequently, we decided to categorize the hospitals based on ICU level [14].

In the western part of The Netherlands, there are a total of 18 hospitals. We approached 17 hospitals and a total of 11 (61 %) wanted to participate in our study. Each participating ED was visited two to four times, to include an approximate 50 % of the total ED nurses employed at each hospital. One of our investigators performed all ED visits. During each visit, all participants were informed about the study, both in writing and verbally. The informed consent was signed by both the participant and the investigator. Hereby, the participants agreed with their participation in this study completely voluntarily and sufficiently informed. After signing the informed consent, the participants were asked to immediately fill in the questionnaire. Our investigator was at the site the whole time, to make sure no communication between participants took place and no information source was used.

Study design, including coding procedure intended to facilitate follow-up measurement, was approved by the NVMO-ERB [15]. The coding procedure contains assignation of a unique code to each individual nurse. This procedure ensures the results being processed anonymously; scores are not traceable to a certain person.

### Methods and measurements

To evaluate the knowledge of ED nurses about SIRS and sepsis, a 35-question form was created. Twenty-nine questions tested knowledge of SIRS and the different stages of sepsis. These questions were categorized: general, protocol, awareness about SIRS, sepsis, severe sepsis, septic shock, treatment and case studies. Factors that may affect the degree of knowledge about SIRS and sepsis among ED nurses were also explored. The other six questions provided demographic information about each participant. With this information, subgroups were formed. This allowed classification of the following subgroups and factors concerning the participants: gender; number of years working as a certified ED nurse; if still in training, the number of months in training; age category; additionally trained for ICU/cardiac care unit (CCU); recent education in the last year concerning SIRS/sepsis; and general hospital and/or academic center work experience and participation during night or day shift. The questionnaire was validated by an expert panel including three medical specialists from the intensive care, an emergency physician, an ED nurse and an educational professional all considered experts concerning either sepsis or questionnaires.

All variables and questions were coded and managed using a code-log. All forms were manually entered into a database. Primary outcome is the amount of points scored on the questionnaire. Each correct answer counts up to a total score with a maximum of 29.

All ED nurses or ED nurses in training who were at work during our visits were asked to participate. No exclusion criteria were defined. At each ED unit, the manager was interviewed to determine characteristics of the ED. The following subgroups were formed: ICU level 1, 2 or 3; number of examination rooms at the ED; total number of patient visits during a whole year; presence of a sepsis protocol (if present, what kind of protocol); and participation during first, second, third or fourth visit.

### Outcomes

The primary outcome measure is the score obtained from the validated question form. Secondary outcome measures are the different factors that contribute to the recognition of SIRS and sepsis.

### Statistical analysis

We used IBM SPSS 20 to process data.

Reliability analysis, to determine the internal consistency of our questionnaire, was evaluated by Cronbach’s alpha. Only the 29 questions with a defined correct answer were included in this analysis. Cronbach’s alpha was 0.526.

Standard descriptive statistics were used to explore our data. One-way ANOVA tests using post hoc Bonferroni test were performed to evaluate differences between subgroups. At last, a linear regression model was computed. Variables which have shown a *p* value <0.20 were included in this model. *p* values <0.05 were defined as significant. Missing answers were handled as ‘system-missing’ data.

## Results

### Characteristics of study subjects

A total of 216 ED nurses participated in our survey, distributed over 11 different hospitals. We achieved a mean sample size of 53.9 % (31.4–91.7 %) of the total number of nurses employed at each hospital. Two nurses refused to participate, one because of personal reasons and the other because of high working load at the time. This resulted in a response rate of 99.1 %.

The general characteristics of the study population are presented in Table 1.

**Table 1:** Demographic data emergency department nurses

Variable		*n* (%)
Sex	Male	53 (25)
	Female	163 (75)
Age (years)	20–30	49 (23)
	31–40	71 (33)
	41–50	45 (21)
	51–65	49 (23)
	Unknown	2 (0)
Shift	Day	182 (84)
	Night	34 (16)
Recent education	Yes	58 (27)
	No	158 (73)
Visit	1	87 (40)
	2	75 (35)
	3	49 (23)
	4	5 (2)
Education	ED	151 (70)
	ED + ICU/CCU	54 (25)
	Unknown	11 (5)
Experience (years)	0–2	72 (33)
	3–11	68 (32)
	≥12	71 (33)
	Unknown	5 (2)
ICU level	1	43 (20)
	2	67 (31)
	3	106 (49)
Sepsis protocol	Yes	193 (89)
	No	23 (11)

The majority of the nurses were female (75 %). A total of 34 ED nurses (15.7 %) were participating at the end of their night shift. Fifty-eight ED nurses (26.9 %) completed sepsis education in the last 12 months (recent education). Twenty-five percent of the ED nurses were also certificated as ICU/CCU nurse, aside from ED certification. In one hospital, a sepsis protocol was not present. ICU level 3 hospitals are larger than ICU level 1 or 2 hospitals, resulting in fewer participants in the subgroups ICU 1 and ICU 2.

### Main results

The overall mean score obtained was 15.9 (SD 3.21, Table 2).

**Table 2:** Emergency department nurses mean scores in association with variables (ANOVA)

Variable		*n* (%)	Mean score (SD)	*p* value
Total		216	15.9 (3.2)	
Sex	Male	53 (24)	16.7 (3.5)	0.04
	Female	163 (76)	15.7 (3.0)	
Age (years)	20–30	49 (23)	16.6 (3.4)	0.02
	31–40	71 (33)	16.3 (3.2)	
	41–50	45 (21)	16.0 (3.3)	
	51–65	51 (23)	14.8 (2.7)	
Shift	Day	182 (84)	15.9 (3.2)	0.57
	Night	34 (16)	16.1 (3.3)	
Recent education	Yes	58 (27)	17.2 (3.3)	0.001
	No	158 (73)	15.5 (3.1)	
Visit	1	87 (40)	16.1 (3.3)	0.43
	2	75 (35)	15.7 (3.2)	
	3	49 (23)	16.2 (2.9)	
	4	5 (2)	13.8 (4.4)	
Education	ED	151 (70)	15.9 (3.3)	0.55
	ED + ICU/CCU	54 (25)	16.2 (3.0)	
	Unknown	11 (5)	15.0 (2.9)	
Experience (years)	0–2	72 (33)	16.5 (3.2)	0.25
	3–11	68 (32)	15.6 (3.4)	
	≥12	71 (33)	15.7 (3.0)	
	Unknown	5 (2)	15.2 (2.4)	
ICU level	1	43 (20)	14.4 (2.8)	0.0001
	2	67 (31)	15.4 (3.2)	
	3	106 (49)	16.9 (3.1)	
Sepsis protocol	Yes	193 (89)	16.1 (3.3)	0.07
	No	23 (11)	14.7 (2.5)	

Gender (*p* = 0.04), age (*p* = 0.02), recent education (*p* < 0.001) and ICU level (*p* < 0.0001) influenced mean scores significantly. Night or day shift, number of site visits, the presence of a sepsis protocol, ICU/CCU experience and number of years ED working experience did not contribute significantly to the obtained mean scores.

The effect of gender is not considered significant (*p* value = 0.066) after correction for age, recent education, ICU level and availability of a sepsis protocol. Age (*p* = 0.001), recent education (*p* = 0.041) and working at a level 3 ICU hospital (*p* = 0.001) are considered significant after correction. Working at an ICU level 2 and availability of a sepsis protocol are not significantly associated with total score when corrected for gender, age, recent education and ICU level 3.

In addition to the linear regression model, we computed a table with all subgroups, including numbers per group and mean total score (Table 3). This data shows mean scores of all subgroups within the different ICU level hospitals.

**Table 3:** Mean scores by group, within ICU level

Variable		ICU level								
		ICU 1			ICU 2			ICU 3		
		*n (*%) within ICU 1	Mean score (SD)	*p*	*n* (%) within ICU 2	Mean score (SD)	*p*	*n* (%) within ICU 3	Mean score (SD)	*p*
ED nurses	Total	43 (100)	14.4 (2.8)		67 (100)	15.4 (3.2)		106 (100)	16.9 (3.1)	
Hospital	Count	3 (27.3)			4 (36.4)			4 (36.4)		
Sex	Male	5 (11.6)	16.0 (2.9)		15 (22.4)	16.1 (3.6)		33 (24.5)	17.1 (3.6)	
	Female	38 (88.4)	14.2 (2.7)	0.22	52 (77.6)	15.2 (3.1)	0.30	73 (68.9)	16.8 (2.9)	0.63
Age (years)	20–30	9 (21.4)	14.9 (2.2)		17 (25.4)	15.8 (3.5)		23 (21.9)	17.9 (3.3)	
	31–40	7 (16.7)	14.6 (1.6)	1	31 (46.3)	15.6 (3.4)	1	33 (31.4)	17.3 (2.9)	1
	41–50	13 (31.0)	15.0 (3.2)	1	9 (13.4)	14.9 (3.3)	1	23 (21.9)	17.2 (3.1)	1
	51–65	13 (31.0)	13.5 (3.2)	1	10 (14.9)	14.6 (1.9)	1	26 (24.8)	15.4 (2.6)	0.03
Shift	Night	6 (14.0)	13.5 (1.4)		15 (22.4)	15.7 (3.3)		13 (12.3)	17.8 (3.0)	
	Day	37 (86.0)	14.5 (2.9)	0.44	52 (77.6)	15.3 (3.2)	0.64	93 (87.7)	16.8 (3.1)	0.27
Recent	Yes	3 (7.0)	16.7 (2.5)		12 (17.9)	16.3 (3.8)		43 (40.6)	17.4 (3.3)	
Education	No	40 (93.0)	14.3 (2.7)	0.18	55 (82.1)	15.2 (3.1)	0.245	63 (59.4)	15.5 (3.1)	0.13
Visit	1	17 (39.5)	14.4 (3.0)		30 (44.8)	15.0 (2.8)		40 (37.7)	17.7 (3.1)	
	2	13 (30.2)	14.3 (3.2)	1	20 (29.9)	15.6 (3.7)	1	42 (39.6)	16.2 (2.9)	0.07
	3	13 (30.2)	14.5 (2.0)	1	17 (25.4)	15.9 (3.3)	0.91	19 (17.9)	17.5 (2.6)	1
	4	–	–	–	–	–	–	5 (4.8)	13.8 (4.4)	0.04
Sepsis	Yes	43 (100)	14.4 (2.8)		44 (65.7)	15.8 (3.5)		106 (100)	16.9 (3.1)	
Protocol	No	0 (0)	–	–	23 (34.3)	14.7 (2.5)	0.15	0 (0)	–	–
Education	ED	33 (80.5)	14.2 (2.8)		49 (73.1)	15.3 (3.2)		69 (71.1)	17.1 (3.2)	
	ED + ICC/CCU	8 (19.5)	15.3 (2.9)	0.41	18 (26.9)	15.7 (3.3)	0.61	28 (28.9)	16.7 (2.8)	0.53
Experience (years)	0–2	10 (25.0)	15.0 (2.2)		27 (40.9)	16.2 (3.4)		35 (33.3)	17.3 (3.2)	
	3–11	18 (45.0)	13.7 (3.2)	0.88	24 (36.4)	15.0 (3.4)	0.56	26 (24.8)	17.4 (2.7)	1
	≥12	12 (30.0)	15.1 (2.7)	1	15 (22.7)	14.4 (2.0)	0.22	44 (41.9)	16.3 (3.2)	0.48

**Fig. 1:**
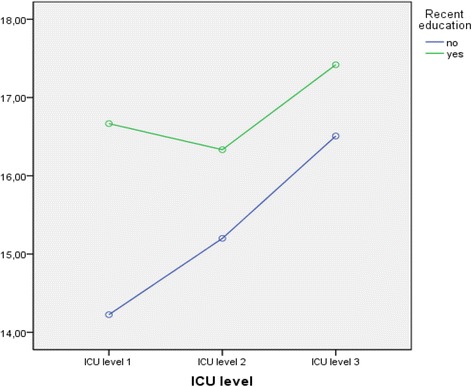
Mean score based on intensive care unit level and recent education

Figure 1 illustrates the effect of the differences in ICU levels and the effect of recent education. Note that, due to the small numbers, ICU levels 1 and 2 are combined into one group. The non-recent educated group ED nurses employed at ICU level 1 or 2 hospitals scored significantly lower than the non-recent educated group ED nurses employed at an ICU level 3 hospital. Comparison of the ED nurses who did report recent education, though, shows no significant difference between the ICU level 1 and 2 group compared to the ICU level 3 group (Table 4).

**Table 4:** Intensive care unit levels and recent education

Recent education	ICU level 1 + 2	ICU level 3	*p* value
	Mean score (SD)	Mean score (SD)	
No	14.8 (3.0)	16.5 (3.0)	0.001
Yes	16.4 (3.5)	17.4 (3.2)	0.267

## Discussion

### Key results

Our study showed that the knowledge of ED nurses concerning SIRS and sepsis rises proportionally with the level of ICU in hospitals. Recent education in sepsis raises knowledge level as well while knowledge seems to decrease with age. Knowledge was not affected by working in the day or night shift or level of ICU hospital when recently educated.

### Limitations

Potential bias could be the multiple visits that were done to include an approximate 50 % of the total ED nurses employed at each hospital. Though when we examined the results comparing visits 1, 2, 3 and sometimes 4, there was no significant growth in knowledge. This suggests that the multiple visits did not create a bias.

### Interpretation

In a recently published study, in-hospital mortality increases in patients with severe sepsis and septic shock when there is a delay in first antibiotic administration [6]. Prompt recognition of SIRS and the sequel of sepsis makes early treatment possible and reduces mortality significantly. For clinical practice, it is important to know what factors influence the level of knowledge among the often first person to see a patient, the ED nurse, as a prompt recognition of sepsis by the ED nurse may increase speed of treatment.

An important finding in this study is that ED nurses’ knowledge concerning SIRS and sepsis without recent education rises proportionally with the level of ICU in hospitals. We hypothesize that this is because the higher ICU level hospitals have more experience with septic patients. Therefore, our findings seem to suggest that experience with septic patients is associated with more knowledge about SIRS and sepsis.

ED nurses who reported recent education scored higher than those who did not report recent education. Therefore, we conclude that education is desirable at a regular base. We also conclude that recent education programs have a positive association with knowledge about SIRS and sepsis.

At the hospitals with ICU level 1 or 2, 18 % of ED nurses reported recent education. In spite of a lower exposure rate to septic patients in the ED, this group did not score significantly lower than nurses working at a hospital with a level 3 ICU. It seems that education is a successful instrument to increase the knowledge level, even when exposure to septic patients is low. Therefore, a good educational program about SIRS and sepsis can compensate for a low level of exposure to septic patients.

Another aspect of our study shows that ED nurses over the age of 50 scored significantly lower than their younger colleagues. We suggest this difference is due to the fact that these ED nurses were certificated before the SIRS criteria where introduced in 1992 [7]. Our study suggests the importance and need of updating courses in this specific group of older nurses.

Some ED nurses participated at the end of their night shift. We assumed a possible negative effect of being tired based on previously published articles [16, 17]; however, both day and night shifts scored approximately the same (15.9 vs 16.1). In this study, knowledge about SIRS and sepsis was not influenced by participation in a day or night shift.

Our study is the first to investigate what factors contribute to the knowledge of SIRS and sepsis in ED nurses. Moreover, a validated questionnaire was used and a large study sample (216 nurses) with a high response rate (99.1 %) was included. In a previous study by Robson et al. [13], ward nurses scored low when they were tested on knowledge of sepsis definitions and its initial management. However, this study was not conducted in the ED, the questionnaire was not validated and more importantly, this study does not answer the question what factors contribute to the level of knowledge. Other studies [9–12] included physicians or residents and were also not focused on factors influencing knowledge of sepsis and SIRS [9]. While it is important to know what the level of knowledge is, it is perhaps even more important to know how this knowledge is influenced.

## Conclusions

In summary, based on this study, working at a hospital with more exposure to sepsis (level 3 ICU), recent education in SIRS and sepsis and younger age are factors associated with higher knowledge about SIRS and sepsis among ED nurses. Working the day or night shift does not influence knowledge about SIRS and sepsis. In our study, a good educational program about SIRS and sepsis seems to compensate for a low level of exposure to septic patients.

## Abbreviations

CCU, cardiac care unit; ED, emergency department; ICU, intensive care unit; NVMO-ERB, Nederlands Vereniging voor Medisch Onderwijs (Dutch society of medical education) Ethical Review Board; PaCO_2_, arterial carbon dioxide tension; SIRS, systemic inflammatory response syndrome

## References

[1] Levy MM, Dellinger RP, Townsend SR (2010). The Surviving Sepsis Campaign: results of an international guideline-based performance improvement program targeting severe sepsis. Intensive Care Med.

[2] Marshall JC, Dellinger RP, Levy M (2010). The Surviving Sepsis Campaign: a history and a perspective. Surg Infect.

[3] Kumar A, Roberts D, Wood KE (2006). Duration of hypotension before initiation of effective antimicrobial therapy is the critical determinant of survival in human septic shock. Crit Care Med.

[4] Talmor D, Greenberg D, Howell MD (2008). The costs and cost-effectiveness of an integrated sepsis treatment protocol. Crit Care Med.

[5] Gao F, Melody T, Daniels DF (2005). The impact of compliance with 6-hour and 24-hour sepsis bundles on hospital mortality in patients with severe sepsis: a prospective observational study. Crit Care.

[6] Ferrer R, Martin-Loeches I, Philips G (2014). Empiric antibiotic treatment reduces mortality in severe sepsis and septic shock from the first hour: results from a guideline-based performance improvement program. Crit Care Med.

[7] Bone RC, Balk RA, Cerra FB (1992). Definitions for sepsis and organ failure and guidelines for the use of innovative therapies in sepsis. The ACCP/SCCM Consensus Conference Committee. American College of Chest Physicians/Society of Critical Care Medicine. Chest.

[8] Tromp M, Hulscher M, Bleeker-Rovers CP (2010). The role of nurses in the recognition and treatment of patients with sepsis in the emergency department: a prospective before-and-after intervention study. Int J Nurs Stud.

[9] Burney M, Underwood J, McEvoy S (2012). Early detection and treatment of severe sepsis in the emergency department: identifying barriers to implementation of a protocol-based approach. J Emerg Nurs.

[10] Ziglam HM, Morales D, Webb K (2006). Knowledge about sepsis among training-grade doctors. J Antimicrob Chemother.

[11] Assuncao M, Akamine N, Cardoso GS (2010). Survey on physicians’ knowledge of sepsis: do they recognize it promptly?. J Crit Care.

[12] Poeze M, Ramsay G, Gerlach H (2004). An international sepsis survey: a study of doctors’ knowledge and perception about sepsis. Crit Care.

[13] Robson W, Beavis S, Spittle N (2007). An audit of ward nurses’ knowledge of sepsis. Nurs Crit Care.

[14] Peelen L, de Keizer NF, Peek N (2007). The influence of volume and intensive care unit organization on hospital mortality in patients admitted with severe sepsis: a retrospective multicentre cohort study. Crit Care.

[15] NVMO Ethical Review Board 2012. Available from: http://www.nvmo.nl/.

[16] Dula DJ, Dula NL, Hamrick C (2001). The effect of working serial night shifts on the cognitive functioning of emergency physicians. Ann Emerg Med.

[17] Grossman VG (1997). Defying circadian rhythm: the emergency nurse and the night shift. J Emerg Nurs.

